# Multifunctional Lipid Nanoparticles Remodeling Tumor Immune Microenvironment for Breast Cancer Chemo-Immunotherapy

**DOI:** 10.32604/or.2026.078278

**Published:** 2026-05-21

**Authors:** Wei Jiang, You Zheng, Zhouhong Jing, Xiangling Yu, Huiying Fang

**Affiliations:** 1 Department of Radiology, The Second Affiliated Hospital of Chongqing Medical University, No. 74 Linjiang Rd, Yuzhong District, Chongqing, China; 2 Department of Breast Cancer Center, Chongqing Key Laboratory for Intelligent Oncology in Breast Cancer (iCQBC), Chongqing University Cancer Hospital, No. 181 Hanyu Rd, Shapingba District, Chongqing, China

**Keywords:** Breast cancer, liposomes, doxorubicin, resiquimod, immunotherapy, chemotherapy, drug delivery

## Abstract

**Background:** Breast cancer treatment is often hampered by the immunosuppressive tumor microenvironment (TME). To improve therapeutic efficacy, this study developed a folic acid-chitosan (FA-CS)-modified liposomal formulation co-delivering doxorubicin (DOX) and resiquimod (R848) for combined chemotherapy and immune modulation. **Methods:** The FA-CS-R848/DOX@Lip liposomes were prepared by rotary evaporation and characterized for morphology, particle size, zeta potential, drug encapsulation efficiency (EE), drug loading (DL) capacity, and drug release profiles. Cellular uptake and cytotoxicity were determined to assess the biological effects of the formulation. Antitumor efficacy and biosafety were assessed in an EO771 tumor-bearing mouse model. The macrophage phenotype, TME composition, and CD8^+^ T cell cytotoxic function were evaluated by flow cytometry. **Results:** The FA-CS-R848/DOX@Lip exhibited a relatively uniform spherical morphology, with an average size of 221.53 nm and a zeta potential of 5.06 mV. The EE (%) of R848 and DOX were over 70% and 80%, respectively, while the DL (%) capacities were 4.6% and 3.1%. Drug release studies showed a slow release profile. *In vitro*, FA-CS-R848/DOX@Lip showed greater cytotoxicity than non-targeted liposomes (*p* < 0.01) and higher uptake in EO771 cells and RAW264.7 macrophages. *In vivo*, treatment with FA-CS-R848/DOX@Lip significantly inhibited tumor growth compared with saline (*p* < 0.0001) without causing significant hematological or biochemical toxicity (*p* > 0.05). In addition, FA-CS-R848/DOX@Lip increased the MHC II/CD206 ratio of tumor-associated macrophages (TAMs), elevated the proportions of CD8^+^ and CD4^+^ T cells, reduced the proportion of myeloid-derived suppressor cells (MDSCs), and enhanced the percentages of IFNγ^+^ and Ki67^+^ CD8^+^ T cells *in vivo*. **Conclusions:** FA-CS-R848/DOX@Lip exerts potent antitumor activity with favorable biosafety *in vivo*. Its therapeutic effect may be associated with improved immune activation and remodeling of the TME, supporting its potential as a chemo-immunotherapeutic strategy for breast cancer.

## 1 Introduction

Breast cancer remains one of the most prevalent and life-threatening malignancies among women worldwide. According to GLOBOCAN 2022, approximately 2.3 million new breast cancer cases were diagnosed globally, accounting for 11.6% of all newly diagnosed cancers and ranking second in global cancer incidence [1]. Moreover, breast cancer continues to be one of the leading causes of cancer-related mortality in women [1]. In China, it similarly ranks as the second most common malignancy, with an estimated 357,200 new cases reported in 2022 [2]. Despite remarkable advances in surgery and adjuvant therapies, the prognosis for patients with advanced or refractory recurrent breast cancer remains suboptimal. These challenges largely stem from the complex and heterogeneous tumor microenvironment (TME), which critically limits therapeutic efficacy. As a complex milieu supporting tumor survival, the TME, particularly its key components, such as immunosuppressive tumor-associated macrophages (TAMs), play a crucial role in mediating immune evasion and therapeutic resistance [3,4]. Therefore, the development of novel therapeutic strategies that can simultaneously target tumor cells and modulate the immunosuppressive TME represents a promising direction for improving breast cancer outcomes.

The TME profoundly influences tumor progression and immune regulation. Among the various immune cells within the TME, TAMs are the most abundant and functionally diverse. These macrophages exhibit phenotypic plasticity, with M1-type macrophages displaying pro-inflammatory and antitumor properties, whereas M2-type TAMs promote tumor progression, immune escape, and poor clinical outcomes [5,6]. M2 macrophages facilitate tumor growth through angiogenesis, inhibition of T-cell activation, and enhancement of tumor invasiveness [7,8]. In addition, TAMs contribute significantly to drug resistance in breast cancer [9,10]. For instance, CCL2 secreted by TAMs activates the PI3K/Akt/mTOR signaling pathway in breast cancer cells, thereby promoting chemoresistance and recruiting additional TAMs. Conversely, drug-resistant tumor cells secrete TNF-α, which triggers the mTORC1-FOXK1 signaling axis in TAMs, driving their polarization toward the M2 phenotype and further amplifying CCL2 secretion to form a positive feedback loop [11]. Hence, reprogramming M2 macrophages toward an M1-like phenotype offers a promising strategy to enhance antitumor immunity and overcome therapeutic resistance.

Resiquimod (R848), a small-molecule agonist of Toll-like receptors 7 and 8 (TLR7/8), has been shown to reprogram immunosuppressive myeloid cells by driving M2-like macrophages toward an M1 phenotype and promoting the differentiation of MDSCs toward an APC-like phenotype, thus contributing to the reversal of the immunosuppressive TME [12,13]. However, its clinical application is hindered by poor water solubility, low systemic bioavailability, and dose-limiting systemic toxicities. Systemic administration can trigger excessive innate immune activation and rapid cytokine release, increasing the risk of systemic inflammatory responses [14], while adverse events such as fever and hematologic toxicity further constrain dosing and treatment schedules [15,16,17]. In addition, the lack of tumor-targeting capability results in off-target immune stimulation and potential tissue injury in healthy organs, and rapid systemic clearance prevents sufficient drug accumulation at the tumor site, collectively limiting its therapeutic potential. Therefore, achieving precise and efficient delivery of immune modulators to the tumor site remains a critical challenge in cancer immunotherapy.

Doxorubicin (DOX) is a widely used chemotherapeutic agent in breast cancer treatment, capable of inducing DNA damage, disrupting cell proliferation, and triggering immunogenic cell death (ICD) [18,19]. ICD facilitates the release of damage-associated molecular patterns (DAMPs), including calreticulin, ATP, HMGB1, and heat shock proteins, which promote tumor antigen recognition and presentation, thereby eliciting robust antitumor immune responses [20,21,22]. Furthermore, DOX stimulates the secretion of pro-inflammatory cytokines such as TNF-α, IL-6, and IL-1β, amplifying immune activation [18]. Consistent with this rationale, the KEYNOTE-522 Japan subgroup analysis further supported the clinical benefit of chemo-immunotherapy in early-stage TNBC, showing a higher pathological complete response (pCR) rate and improved long-term event-free survival with pembrolizumab plus neoadjuvant chemotherapy (followed by adjuvant pembrolizumab) compared with chemotherapy alone [23]. Consequently, combining chemotherapy and immunotherapy has emerged as a synergistic approach that not only induces tumor cell death but also enhances immune-mediated tumor eradication.

Recent advances in nanotechnology have opened new avenues for cancer therapy by improving drug bioavailability, tumor accumulation, and retention time. Among various nanocarriers, liposomal nanoparticles have attracted extensive interest due to their biocompatibility and capacity to encapsulate both hydrophilic and hydrophobic agents. Nevertheless, conventional liposomes often suffer from limited targeting specificity and premature drug release [24]. To address these challenges, the present study designed a novel lipid-based nanoplatform featuring a chitosan (CS) coating to enhance liposomal stability and bioavailability [25,26]. Moreover, surface functionalization with folic acid (FA) enables active targeting of tumor cells and TAMs via folate receptor-mediated uptake [27]. This dual-targeting, dual-drug delivery system co-encapsulates DOX and R848, integrating the cytotoxic effects of DOX with the immune-reprogramming activity of R848. By simultaneously inducing tumor cell apoptosis and reactivating antitumor immunity, the system aims to achieve potent synergistic therapeutic efficacy. In summary, this study proposes a multifunctional nanoplatform that unites chemotherapy and immunotherapy through precise co-delivery of DOX and R848, offering a promising strategy for overcoming current limitations in breast cancer treatment and advancing the development of integrated cancer therapeutics.

## 2 Materials and Methods

### 2.1 Materials

Soy lecithin (Aladdin, Shanghai, China; Cat. No. L105732); 1-ethyl-3-(3-dimethylaminopropyl)-carbodiimide hydrochloride (EDC.HCl; Cat. No. E7750), N-hydroxysuccinimide (NHS; Cat. No. 130672), cholesterol (Chol; Cat. No. C8667), low molecular weight CS (Cat. No. 448869), and FA (Cat. No. F7876) were purchased from Sigma-Aldrich (St. Louis, MO, USA); R848 (Selleck, Houston, TX, USA; Cat. No. S8133); DOX (MCE, Monmouth Junction, NJ, USA; Cat. No. HY-15142); Other reagents, such as chloroform (Cat. No. C1506328), methanol (Cat. No. M116115), and ethanol (Cat. No. E111992), were supplied by Aladdin (Shanghai, China).

The murine mammary carcinoma cell line (EO771) and murine macrophage cell line (RAW264.7) were obtained from the American Type Culture Collection (ATCC; Manassas, VA, USA). Both cell lines have been STR-identified and tested negative for mycoplasma contamination. They were cultured in Dulbecco’s Modified Eagle’s Medium (DMEM; Gibco, Grand Island, NY, USA) supplemented with 10% fetal bovine serum (FBS; Gibco, Grand Island, NY, USA) in a humidified incubator at 37°C with 5% CO_2_. 

### 2.2 Construction of Targeted Drug-Loaded Liposomes

#### 2.2.1 Preparation of R848/DOX@Liposomes

Liposomes were prepared using the thin-film hydration method [28]. Soy lecithin (15 mg), cholesterol (3 mg), and R848 (2 mg) were dissolved in 6 mL of dichloromethane: methanol (v/v = 2:1). The solution was sonicated (Aladdin, Shanghai, China; Cat. No. S6103-04-13L) for 2 min, and the solvent was removed by rotary evaporation at 40°C, followed by nitrogen flushing for 5 min to form a lipid film. The film was vacuum-dried for 8 h and then hydrated with 300 mM citric acid buffer for 30 min. The resulting suspension was extruded through 0.4 μm and 0.2 μm polycarbonate membranes to obtain liposomes.

The liposomal suspension was mixed with a 300 mM citric acid–doxorubicin buffer (drug-to-lipid ratio = 1:20), and the pH was adjusted to 7.5 with Na_2_CO_3_. After incubation for 30 min, DOX was encapsulated into the liposomes. Blank liposomes (Lip), R848-loaded liposomes (R848@Lip), and DOX-loaded liposomes (DOX@Lip) were prepared using the same procedure. Unencapsulated drugs were removed by gel filtration, and the liposomes were stored at 4°C.

#### 2.2.2 Preparation of FA-CS-R848/DOX@Lip

Low molecular weight CS (100 mg) was dissolved in 1% acetic acid and filtered to remove insoluble residues. FA (44.1 mg), EDC.HCl (38.2 mg), and NHS (23 mg) were dissolved in 10 mL of DMSO, stirred for 1 h, and then added dropwise to the CS solution. The reaction mixture was stirred at room temperature for 16 h. The pH was adjusted to 9.0 with 1 M NaOH, and the resulting FA-CS conjugate was collected by centrifugation (Centrifuge 5804, Eppendorf SE, Hamburg, Germany) at 12,000 rpm for 15 min. The precipitate was washed three times with sodium bicarbonate buffer (pH 8.5), dialyzed (10 kDa MWCO) against PBS (pH 7.4; 4°C) for 3 days, and lyophilized to obtain FA-CS powder.

To prepare FA-CS-R848/DOX@Lip, FA-CS powder (25 mg) was dissolved in 1% acetic acid to obtain a 0.25% (w/v) solution, which was added dropwise to the liposome suspension. After stirring at room temperature for 1 h, the mixture was stored overnight at 4°C, filtered through a 200 nm membrane for sterilization, and aliquoted. FA-CS-R848@Lip and FA-CS-DOX@Lip were prepared following the same procedure.

### 2.3 Characterization of Targeted Drug-Loaded Liposomes

#### 2.3.1 FA-CS Conjugate Characterization

FA was dissolved in DMSO-d6. CS and the FA-CS conjugates were dissolved in a mixture of D_2_O and DCl (35 wt% DCl in D_2_O) to achieve a final DCl concentration of approximately 1% (v/v). The proton nuclear magnetic resonance (^1^H NMR) spectra of the samples were subsequently recorded using a 600 MHz NMR spectrometer (Agilent Technologies, Santa Clara, CA, USA).

#### 2.3.2 Morphology, Size, and Zeta Potential of Liposomes

The morphology of R848/DOX@Lip and FA-CS-R848/DOX@Lip was observed by transmission electron microscopy (TEM; FEI Tecnai G2 spirit, Hillsboro, OR, USA) after negative staining with 2% phosphotungstic acid (Aladdin, Shanghai, China; Cat. No. E1510324). Particle size distribution and zeta potential were measured using dynamic light scattering (DLS; Zetasizer Nano ZS90; Malvern Panalytical, Malvern, Worcestershire, UK) at 25°C, with each sample measured in triplicate. These measurements were used as the baseline values (day 0) for the subsequent storage stability study.

#### 2.3.3 Storage Stability of Liposomes

Liposome dispersions were stored at 4°C in the dark for 4 weeks. Samples were collected on days 0, 7, 14, 21, and 28, and particle size, PDI, and zeta potential were re-measured by DLS under the same conditions as described above.

#### 2.3.4 Encapsulation Efficiency (EE) and Drug Loading (DL) of DOX and R848 in R848/DOX@Lip and FA-CS-R848/DOX@Lip

An aliquot (1 mL) of FA-CS-R848/DOX@Lip suspension was applied dropwise onto a mini gel filtration column (Sephadex G-25, GE Healthcare, Chicago, IL, USA) and centrifuged at 4000 rpm for 10 min to collect the liposome fraction (encapsulated drug). The column was then washed three times with 2 mL of double-distilled water under the same conditions, and the wash fractions were combined as the free-drug fraction. Methanol was added to each sample to achieve a final methanol content of 80% (v/v), followed by sonication (bath) to disrupt liposomes and extract drugs, then centrifugation at 15,000 rpm for 5 min. Supernatants were collected for high-performance liquid chromatography (HPLC; Waters, Milford, MA, USA) analysis using a C18 column (5 μm, 4.6 × 250 mm, Agilent Technologies, Santa Clara, CA, USA) [29]. 

For R848, mobile phase A was 0.1% formic acid in water and mobile phase B was 0.1% formic acid in acetonitrile, with a gradient of 20% B → 95% B → 20% B (re-equilibration), at 1.0 mL/min and 20 μL injection; detection was 254 nm. For DOX, an isocratic mobile phase of methanol/2% (v/v) acetic acid (55/45, v/v) was used at 1.0 mL/min with 20 μL injection; detection was 480 nm.

Encapsulated drug (m_encap) and free drug (m_free) were quantified from the liposome fraction and the free-drug fraction, respectively. EE% and DL% were calculated as:

(1) $$\mathrm{EE}\%=\frac{\text{m\_encap}}{\text{m\_encap}+\text{m\_free}}\times 100\%$$

(2) $$\mathrm{DL}\%=\frac{\text{m\_drug}}{\text{m\_liposome}}\times 100\%$$

where m_liposome was obtained by accurately weighing the FA-CS-R848/DOX@Lip after lyophilization to constant weight. m_drug was determined by disrupting the lyophilized sample with methanol, followed by HPLC quantification of R848 and DOX. The EE (%) and drug DL (%) of R848 and DOX within the R848/DOX@Lip were quantified with the same determination method.

#### 2.3.5 In Vitro Drug Release Kinetics of Liposomes

The *in vitro* release profiles of R848/DOX@Lip and FA-CS-R848/DOX@Lip were evaluated using the dialysis method. Phosphate-buffered saline (PBS, pH 7.4) containing 0.5% (w/w) Tween-80 was used as the release medium. Liposomal dispersions were placed in dialysis bags (MWCO 12–14 kDa) and immersed in 50 mL of release medium at 37°C under gentle stirring. At predetermined intervals (0.5, 1, 2, 4, 8, 16, 24, 36, and 48 h), 1 mL of medium was withdrawn and replaced with an equal volume of fresh buffer. The concentrations of DOX and R848 were determined by HPLC, and cumulative release percentages were calculated.

### 2.4 Cellular Uptake and Cytotoxicity of Targeted Drug-Loaded Liposomes

#### 2.4.1 Cellular Uptake

RAW 264.7 and EO771 cells in the logarithmic growth phase were seeded into glass-bottom dishes at a density of 1 × 10^5^ cells/well and incubated overnight. The cells were then treated with DiI-labeled R848/DOX@Lip or FA-CS-R848/DOX@Lip in DMEM for 4 h. After incubation, the medium was removed, and the cells were washed three times with ice-cold PBS (pH = 7.4), fixed with 4% paraformaldehyde for 10 min, and washed twice with PBS. The cell membrane was stained using DiO working solution (5 μM in PBS) at room temperature for 20 min in the dark. Nuclei were stained with DAPI for 5 min (5 μg/mL) at room temperature for 5 min, followed by PBS washing. Cellular uptake was observed using laser scanning confocal microscopy (LSCM; STELLARIS 5; Leica, Wetzlar, Germany).

#### 2.4.2 Cytotoxicity Assays

Cytotoxicity assays were assessed using the CCK8 assay. EO771 cells were seeded in 96-well plates at 5 × 10^3^ cells/well (100 μL) and cultured to 50%–60% confluence. The cells were then exposed to drug-loaded liposomes at different DOX concentrations (6.25, 12.50, and 25.00 μg/mL), with 10 μL of each formulation added to each well for 24 h and 48 h. After incubation, 10 μL of CCK8 solution (Cat. No. C0037; Beyotime Institute of Biotechnology, Shanghai, China) was added to each well under light protection, and the cells were incubated for an additional 1–2 h. Absorbance was measured at 450 nm (Multiskan^TM^ FC, Thermo Fisher Scientific, Vantaa, Finland), and relative cell viability was calculated. Control experiments were conducted in parallel.

### 2.5 In Vivo Antitumor Efficacy of Liposomes

All animal experiments were approved by the Ethics Committee of the Second Affiliated Hospital of Chongqing Medical University (2023-325). All care of the animals and the experiments conducted on them were carried out in accordance with international animal research guidelines. Female C57BL/6 mice (6–8 weeks old) were purchased from Ensiweier Biotechnology Co. (Chongqing, China), and housed under standard laboratory conditions with free access to food and water. EO771 cells (4 × 10^5^) were inoculated into the second-to-last pair of mammary fat pads on the right side to establish tumor models. EO771 is widely used as an immunocompetent syngeneic breast tumor model (C57BL/6 background), enabling evaluation of therapeutic effects in the context of an intact immune system, which is essential for chemo-immunotherapy studies [30]. 

When tumors reached approximately 100 mm^3^, mice were randomly assigned to two groups (n = 5 per group), with ear tag numbers used for animal identification, and treated with saline or FA-CS-R848/DOX@Lip via tail vein injection (DOX: 5 mg/kg) on day 0, 4, 8, and 12. Tumor volume was monitored every 2–3 days using calipers by an investigator blinded to group allocation. The sample size (n = 5 per group) was determined based on previous experience, preliminary experiments, and commonly used group sizes in similar *in vivo* antitumor studies. Inclusion criteria were successful tumor establishment and tumor volume reaching approximately 100 mm^3^ before treatment initiation. No animals met the exclusion criteria, and no animals were excluded from the final analysis. Mice were euthanized on day 22 after the first treatment.

### 2.6 Analysis of the TME and Macrophage Phenotype

At designated time points post-treatment, mice were euthanized, and tumors were excised, minced, and digested at 37°C for 30 min in serum-free DMEM containing DNase I (Cat. No. 10104159001; Roche, Mannheim, Germany) and Liberase TL (Cat. No. 05401020001; Roche, Mannheim, Germany). The enzymes were first reconstituted in sterile water and then diluted in serum-free DMEM to final working concentrations of 0.33 mg/mL DNase I and 0.25 mg/mL Liberase TL immediately before use. The resulting tissue was filtered through a 70 μm mesh, and red blood cells were lysed using 1× red blood cell lysis buffer prepared from a 10× stock (Cat. No. 130-094-183; Miltenyi Biotec, Bergisch Gladbach, Germany) to obtain single-cell suspensions from tumors. Flow cytometry was performed to analyze immune cell populations within the tumor microenvironment using a CytoFLEX flow cytometer (Beckman Coulter, Brea, CA, USA). Single-cell suspensions from tumors were stained with fluorescently conjugated antibodies. Tumor cell suspensions from tumor-bearing mice were stained with Fixable Viability Dye eFluor^TM^ 450 (Cat. No. 65-0863-14; Invitrogen, Carlsbad, CA, USA) for 15–30 min at 4°C to discriminate live/dead cells. Cells were washed once with flow cytometry buffer to remove unbound dye and then stained with fluorescently labeled antibodies against cell surface markers, including CD45 (Cat. No. 103116), CD11b (Cat. No. 101263), CD3 (Cat. No. 100209), CD8a (Cat. No. 100722, 100706), CD4 (Cat. No. 100433), Gr-1 (Cat. No. 108408), F4/80 (Cat. No. 123114), MHC II (Cat. No. 107626), all from BioLegend (San Diego, CA, USA) and diluted 1:100 in staining buffer for 30 min at 4°C.

For intracellular staining, cells were fixed and permeabilized using the Foxp3/Transcription Factor Staining Buffer Set (Cat. No. 2507021; eBioscience, San Diego, CA, USA), antibodies against CD206 (Cat. No. 141708; BioLegend, San Diego, CA, USA). For cytokine detection, cells were stimulated with Cell Stimulation Cocktail (Cat. No. 00-4975-93; Invitrogen, Carlsbad, CA, USA) at 37°C for 4 h prior to intracellular staining with antibodies against IFN-γ (Cat. No. 505810) and Ki67 (Cat. No. 151211) from BioLegend (San Diego, CA, USA).

### 2.7 In Vivo Safety Evaluation of Liposomes

At the study endpoint, blood was collected by cardiac puncture under deep anesthesia for hematological and biochemical analyses. Following blood collection, the major organs were excised, fixed in 4% paraformaldehyde, embedded in paraffin, and sectioned at 4 μm. Hematoxylin and eosin (H&E) staining was performed by a professional histopathology platform according to standard protocols. Briefly, sections were deparaffinized, rehydrated, stained with hematoxylin and eosin at room temperature, and then dehydrated, cleared, and mounted for histopathological evaluation of the biosafety of FA-CS-R848/DOX@Lip.

### 2.8 Statistical Analysis

All experiments were conducted independently in biological triplicate. Data are presented as mean ± standard deviation (SD). Statistical analyses were performed using IBM SPSS Statistics version 20.0 (IBM Corp., Armonk, NY, USA). Comparisons between two groups were conducted using an unpaired two-tailed Student’s *t*-test, while comparisons among multiple groups were performed using one-way analysis of variance (ANOVA) followed by Tukey’s post hoc test. A two-sided *p* value < 0.05 was considered statistically significant, with significance levels indicated as *p < 0.05, **p < 0.01, ***p < 0.001, and ****p < 0.0001.

## 3 Results

### 3.1 Synthesis of FA-CS

**Figure 1:**
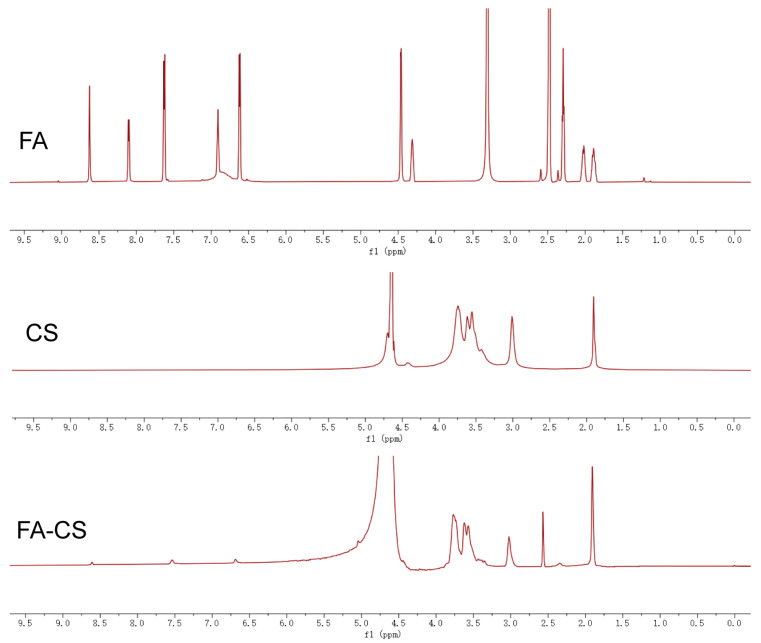
^1^H NMR spectra of FA, CS and FA-CS.

FA and CS were chemically conjugated via carbodiimide coupling to form FA-CS, and characterized by ^1^H-NMR (Fig. 1). The ^1^H-NMR spectrum of CS showed peaks at 3.0, 3.4, 3.5, 3.6, and 3.7 ppm, corresponding to the glucosamine ring of CS. The ^1^H-NMR spectrum of FA exhibited peaks at 6.6, 7.6, and 8.6 ppm, representing the aromatic rings of FA [31]. The ^1^H-NMR spectrum of the FA-CS conjugate displayed overlapping peaks at 2.9, 3.4, 3.5, 3.6, and 3.7 ppm (chitosan glucosamine ring) and at 6.7, 7.5, and 8.6 ppm, confirming the presence of FA in the conjugate.

### 3.2 Characterization of Liposomes: TEM, Particle Size, and Zeta Potential

**Figure 2:**
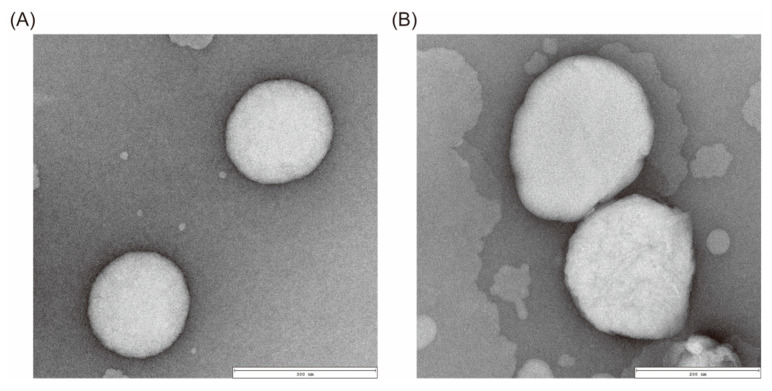
TEM images showing the morphology of liposomal formulations. (**A**) TEM image of R848/DOX@Lip. (**B**) TEM image of FA-CS-R848/DOX@Lip.

TEM images showed that the liposomes were spherical with a relatively uniform size distribution (Fig. 2). Surface modification with FA-CS did not alter the morphology, indicating that the encapsulation of FA-CS in the R848/DOX@Lip liposomes did not affect their structural integrity. Table 1 shows the DLS results, with average particle sizes of R848/DOX@Lip and FA-CS-R848/DOX@Lip measured as (210.47 ± 2.18) nm and (221.53 ± 3.82) nm, respectively. The zeta potential of R848/DOX@Lip was (−22.42 ± 1.17) mV. After surface modification with FA-CS, which carries a positive charge in mildly acidic environments, the zeta potential of FA-CS-R848/DOX@Lip increased to (5.06 ± 0.12) mV. The change in zeta potential confirms the successful surface modification of R848/DOX@Lip with FA-CS.

**Table 1:** Particle size and zeta potential of R848/DOX@Lip and FA-CS-R848/DOX@Lip (n = 3).

Liposomal Formulations	Size (nm)	PDI	Zeta Potential (mV)
R848/DOX@Lip	210.47 ± 2.18	0.18 ± 0.01	−22.42 ± 1.17
FA-CS-R848/DOX@Lip	221.53 ± 3.82	0.25 ± 0.01	5.06 ± 0.12

**Note:** FA-CS, folic acid-chitosan; R848, resiquimod; DOX, doxorubicin; PDI, polydispersity index.

### 3.3 Storage Stability of Liposomes at 4°C

To evaluate storage stability, R848/DOX@Lip and FA-CS-R848/DOX@Lip were stored at 4°C for 4 weeks and characterized on days 0, 7, 14, 21, and 28. As summarized in Table 2, no notable changes were observed in particle size, PDI, or zeta potential for either formulation during storage.

**Table 2:** Storage stability of liposomes at 4°C (n = 3).

Time	R848/DOX@Lip			FA-CS-R848/DOX@Lip		
	Size (nm)	PDI	Zeta Potential (mV)	Size (nm)	PDI	Zeta Potential (mV)
Day 0	210.47 ± 2.18	0.18 ± 0.01	−22.42 ± 1.17	221.53 ± 3.82	0.25 ± 0.01	5.06 ± 0.12
Day 7	211.30 ± 2.52	0.19 ± 0.04	−22.30 ± 1.97	223.27 ± 5.53	0.25 ± 0.02	5.03 ± 0.21
Day 14	212.63 ± 4.18	0.21 ± 0.03	−20.53 ± 1.21	224.77 ± 4.15	0.24 ± 0.02	4.93 ± 0.31
Day 21	214.33 ± 3.25	0.22 ± 0.03	−19.63 ± 1.45	228.33 ± 3.06	0.23 ± 0.03	4.60 ± 0.36
Day 28	220.27 ± 3.52	0.23 ± 0.03	−18.83 ± 1.94	232.67 ± 3.47	0.26 ± 0.02	3.87 ± 0.25

**Note:** FA-CS, folic acid-chitosan; R848, resiquimod; DOX, doxorubicin; PDI, polydispersity index.

### 3.4 EE (%) and DL (%) of R848 and DOX in Liposomes

The EE (%) and DL (%) of R848 and DOX in R848/DOX@Lip and FA-CS-R848/DOX@Lip were determined by HPLC. As shown in Table 3, the EE (%) of DOX in both R848/DOX@Lip and FA-CS-R848/DOX@Lip exceeded 80%, while the EE (%) of R848 was above 70%. These results indicate that surface modification with FA-CS did not significantly affect encapsulation efficiency. The DL (%) of DOX in R848/DOX@Lip and FA-CS-R848/DOX@Lip was (4.5 ± 0.4)% and (3.1 ± 0.5)%, respectively, while that of R848 was (6.8 ± 0.3)% and (4.6 ± 0.2)%, respectively.

**Table 3:** EE (%) and DL (%) of DOX and R848 in R848/DOX@Lip and FA-CS-R848/DOX@Lip (n = 3).

Liposomal Formulations	EE% (DOX)	EE% (R848)	DL% (DOX)	DL% (R848)
R848/DOX@Lip	86.6 ± 1.6	73.7 ± 1.9	4.5 ± 0.4	6.8 ± 0.3
FA-CS-R848/DOX@Lip	83.3 ± 2.1	70.5 ± 2.8	3.1 ± 0.5	4.6 ± 0.2

**Note:** FA-CS, folic acid-chitosan; R848, resiquimod; DOX, doxorubicin; EE, encapsulation efficiency; DL, drug loading.

### 3.5 Drug Release Rate of Liposomes

**Figure 3:**
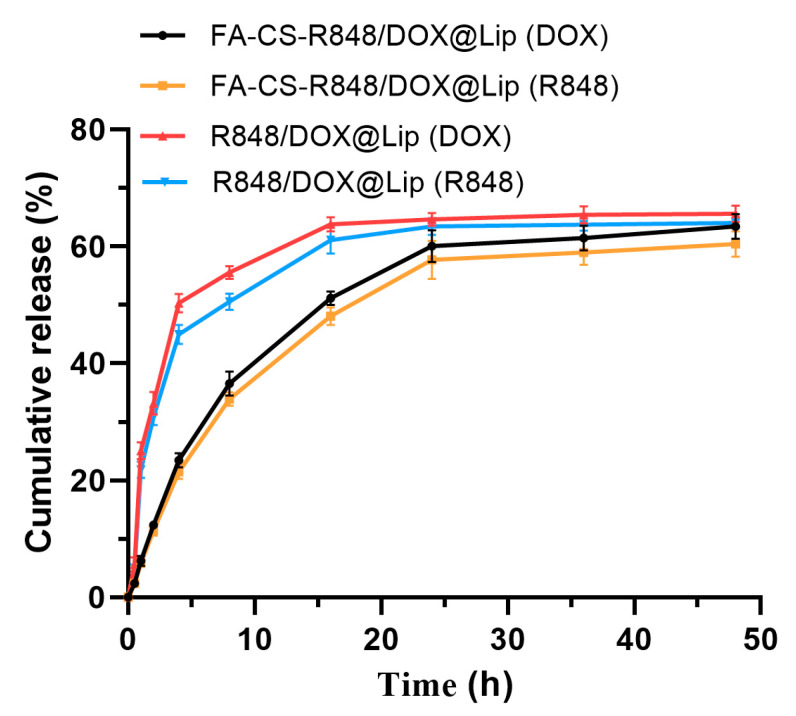
Cumulative release rates of DOX and R848 in R848/DOX@Lip and FA-CS-R848/DOX@Lip (n = 3).

Drug release was evaluated using the dialysis bag method. R848/DOX@Lip and FA-CS-R848/DOX@Lip were placed in dialysis membranes and incubated in PBS (pH 7.4) as the release medium, with the experiment conducted at 37°C and 100 rpm. The *in vitro* release profiles are presented to describe the release behavior of DOX and R848 from the liposomes under the tested conditions. As shown in Fig. 3, compared to R848/DOX@Lip, drug release from FA-CS-R848/DOX@Lip was slower, likely due to the FA-CS coating on the liposome surface.

### 3.6 Cytotoxicity Analysis of Liposomes

**Figure 4:**
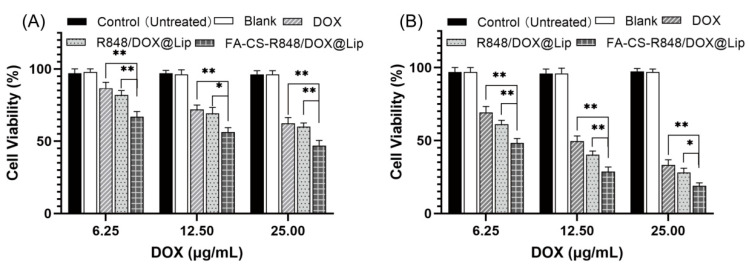
Cytotoxicity of different treatment groups in EO771 cells after 24 and 48 h of incubation (n = 3). (**A**) Cell viability of EO771 cells after 24 h of incubation with different treatment groups. (**B**) Cell viability of EO771 cells after 48 h of incubation with different treatment groups. Data are presented as mean ± SD. *p < 0.05, **p < 0.01.

Cytotoxicity of the liposomes was evaluated using the EO771 cell line. The results showed that blank liposomes exhibited negligible cytotoxicity. At concentrations of 6.25 μg/mL, 12.5 μg/mL, and 25 μg/mL, FA-CS-R848/DOX@Lip demonstrated significantly higher cytotoxicity compared to non-targeted R848/DOX@Lip and free DOX solution. After 48 h of incubation, nearly 80% of the cells treated with FA-CS-R848/DOX@Lip at the highest concentration were dead, indicating markedly enhanced cytotoxicity relative to cells treated with free drug formulations, as shown in Fig. 4.

### 3.7 Targeting of Liposomes to EO771 Cells and RAW 264.7 Cells

**Figure 5:**
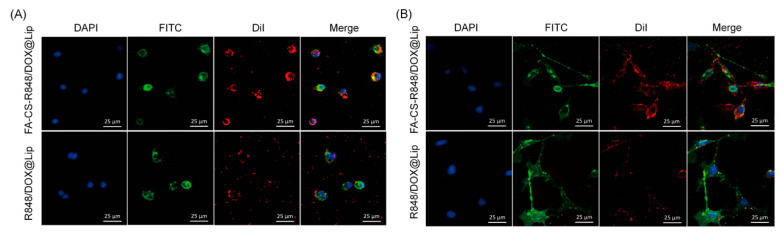
CLSM images showing the targeting and cellular uptake of liposomes in EO771 tumor cells and RAW264.7 macrophages. (**A**) Cellular uptake of R848/DOX@Lip and FA-CS-R848/DOX@Lip in EO771 tumor cells. (**B**) Cellular uptake of R848/DOX@Lip and FA-CS-R848/DOX@Lip in RAW264.7 macrophages. Scale bars = 25 μm.

FA receptors are highly expressed on the surface of both tumor cells and macrophages, making FA modification of nanoparticles an effective strategy for targeted cancer therapy. To evaluate the targeting potential of FA-CS-R848/DOX@Lip to cancer cells and RAW 264.7 macrophages, uptake studies were performed using EO771 breast cancer cells and RAW 264.7 cells. As shown in Fig. 5, in the non-targeted group (R848/DOX@Lip), the red fluorescence signal around the EO771 and RAW 264.7 cell nuclei was weak compared to the blue fluorescence signal. In contrast, the targeted group (FA-CS-R848/DOX@Lip) exhibited significantly stronger red fluorescence signals, indicating that FA-CS modification enhances the drug’s targeting and uptake efficiency.

### 3.8 Inhibition of Tumor Growth by Liposomes in the Mouse Breast Cancer Model

**Figure 6:**
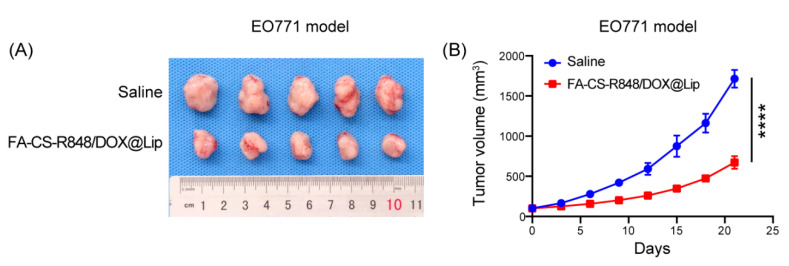
Antitumor effect of FA-CS-R848/DOX@Lip in the EO771 tumor model. (**A**) Photographs of tumors collected from mice treated with saline or FA-CS-R848/DOX@Lip at the end of treatment. (**B**) Tumor growth of EO771 tumor-bearing mice after the indicated treatments. ****p < 0.0001.

To evaluate the inhibitory effect of FA-CS-R848/DOX@Lip on tumor growth, an EO771 breast cancer model was established. As shown in Fig. 6, the FA-CS-R848/DOX@Lip group exhibited significant tumor growth inhibition compared to the saline group. 

### 3.9 Remodeling of the TME and Enhancement of CD8^+^ T Cell Function

**Figure 7:**
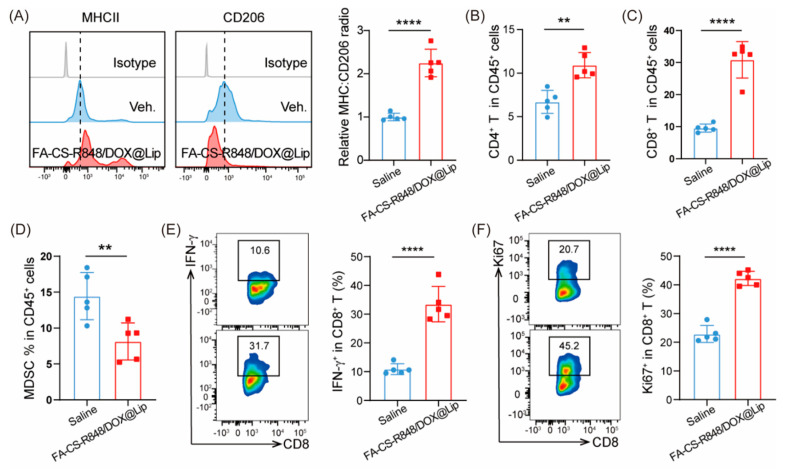
FA-CS-R848/DOX@Lip enhances T cell antitumor immunity. (**A**) MHC II: CD206 ratio of TAMs in EO771 tumor-bearing mice treated with saline or FA-CS-R848/DOX@Lip (n = 5 per mouse group). (**B**–**D**) Percentages of CD4^+^ T cells (**B**), CD8^+^ T cells (**C**), and MDSCs (**D**) in EO771 tumor-bearing mice treated with saline or FA-CS-R848/DOX@Lip (n = 5 per mouse group). (**E**,**F**) Percentage of IFN-γ^+^ (**E**) and Ki67 (**F**) CD8^+^ T cells in EO771 tumor-bearing mice treated with saline or FA-CS-R848/DOX@Lip (n = 5 per mouse group). **p < 0.01, ****p < 0.0001.

As shown in Fig. 7A–F, flow cytometry analysis revealed that the MHC II/CD206 ratio in TAMs was significantly higher in the FA-CS-R848/DOX@Lip group compared to the saline group, indicating a shift toward a pro-immunogenic phenotype in the experimental group TAMs. Analysis of the TME revealed a significant increase in the proportions of CD4^+^ T cells and CD8^+^ T cells in the TME of mice treated with FA-CS-R848/DOX@Lip compared to those in the saline group, while the proportion of MDSCs decreased. This suggests that FA-CS-R848/DOX@Lip effectively remodels the tumor TME, shifting it toward an immune-activated state. Furthermore, the expression levels of IFN-γ and Ki67 in CD8^+^ T cells were elevated, indicating that FA-CS-R848/DOX@Lip enhances the cytotoxic activity and proliferative capacity of CD8^+^ T cells.

### 3.10 In Vivo Biosafety of Liposomes

**Figure 8:**
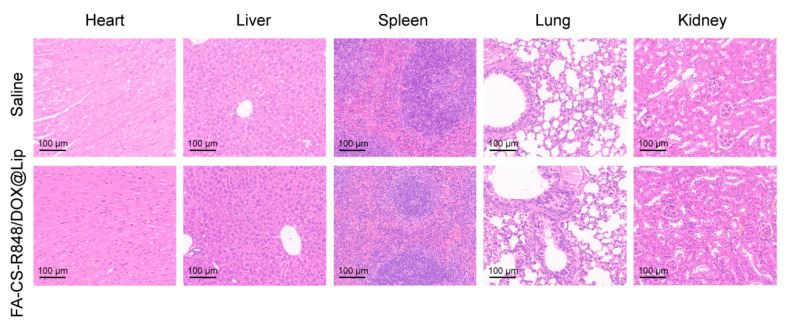
H&E staining of major organs from mice in the saline group and FA-CS-R848/DOX@Lip group. Scale bars = 100 μm.

Biosafety of the nanodrug was assessed through hematological and histopathological analyses in mice. Compared with the saline group, mice treated with FA-CS-R848/DOX@Lip showed no significant changes in blood cell counts or biochemical parameters (Table 4 and Table 5). No statistically significant differences were observed in any parameter compared to the control group (*p* > 0.05). Moreover, histopathological examination revealed no detectable lesions in major organs, indicating that FA-CS-R848/DOX@Lip exhibits excellent biocompatibility and systemic safety (Fig. 8).

**Table 4:** Complete blood count of mice in saline and FA-CS-R848/DOX@Lip groups (n = 5).

Treatment Groups	HGB (g/L)	WBC (×10^9^/L)	PLT (×10^9^/L)	NEU (×10^9^/L)
Saline	140.33 ± 8.96	5.63 ± 0.76	1088.80 ± 180.85	1.43 ± 0.25
FA-CS-R848/DOX@Lip	143.00 ± 5.57	5.70 ± 0.46	1086.33 ± 149.15	1.47 ± 0.15

**Note:** HGB, hemoglobin; WBC, white blood cell count; PLT, platelet count; NEU, neutrophil count; FA-CS, folic acid-chitosan; R848, resiquimod; DOX, doxorubicin.

**Table 5:** Hepatic and renal function of mice in saline and FA-CS-R848/DOX@Lip groups (n = 5).

Treatment Groups	ALT (U/L)	AST (U/L)	TBIL (μmol/L)	BUN (mg/dL)	CR (μmol/L)
Saline	56.47 ± 6.43	169.44 ± 19.77	8.66 ± 0.78	18.69 ± 0.85	30.38 ± 2.90
FA-CS-R848/DOX@Lip	59.67 ± 7.11	140.67 ± 9.14	9.19 ± 0.94	19.33 ± 0.51	33.72 ± 4.77

**Note:** ALT, alanine aminotransferase; AST, aspartate aminotransferase; TBIL, total bilirubin; BUN, blood urea nitrogen; CR, creatinine; FA-CS, folic acid-chitosan; R848, resiquimod; DOX, doxorubicin.

## 4 Discussion

This study successfully developed the FA-CS-R848/DOX@Lip targeted drug delivery system and systematically evaluated its potential application in cancer immunotherapy. By co-encapsulating DOX with the immune activator R848 within folic acid-functionalized chitosan-modified liposomes, this system not only effectively targets tumor cells but also promotes the remodeling of the tumor immune microenvironment through the immunomodulatory action of R848.

In this study, we characterized the key physicochemical attributes of the formulations, including particle size, PDI, and zeta potential, as these parameters critically influence colloidal stability and biological performance. The FA-CS coating led to a slightly increased hydrodynamic diameter relative to the non-coated liposomes, consistent with the presence of an additional polymer/ligand layer on the surface, while maintaining a narrow size distribution, indicating good dispersion uniformity. Importantly, storage at 4°C for 4 weeks did not cause notable changes in particle size or PDI, supporting satisfactory physicochemical stability and suggesting minimal aggregation or fusion during refrigerated storage. Although FA-CS-R848/DOX@Lip exhibited a mildly positive zeta potential (≈+5 mV), polymer-decorated liposomes may maintain colloidal stability primarily through steric hindrance and surface hydration rather than electrostatic repulsion.

In drug delivery, the FA-CS-R848/DOX@Lip system enhanced the drug uptake efficiency in both tumor cells and TAMs through a dual-targeting strategy. Targeting experiments on EO771 and RAW 264.7 cells demonstrated that FA-CS-modified liposomes exhibited significantly stronger fluorescent signals compared to unmodified liposomes, indicating the effectiveness of the folate receptor-targeting strategy. This finding is consistent with existing literature, which suggests that nanoparticles targeting folate receptors can effectively increase drug accumulation in breast cancer cells [32,33]. Additionally, previous studies have shown that positively charged nanoparticles can facilitate the interaction between nanoparticle carriers and negatively charged cancer cells [34]. Consistent with this general principle, EO771 murine breast cancer cells are expected to present a net negative surface charge in physiological media, which may favor electrostatic association with mildly cationic carriers. In this study, FA-CS coating modified the zeta potential of the liposomes, which may enhance the electrostatic interactions between the liposomes and negatively charged cancer cells.

Regarding drug release, the FA-CS-R848/DOX@Lip system demonstrated sustained release properties. According to the release curve, the release rate of FA-CS-R848/DOX@Lip was lower than that of R848/DOX@Lip, with no burst release observed. Even after 48 h, the cumulative release rate of DOX and R848 from FA-CS-R848/DOX@Lip was approximately 60%, indicating its good sustained release capability. This sustained release characteristic allows prolonged drug exposure to tumor cells and immune cells, potentially leading to more effective therapeutic outcomes in the tumor microenvironment [35]. Furthermore, the slower release rate of FA-CS-R848/DOX@Lip may be attributed to the formation of a protective layer by the polymer material on the liposome surface in the release medium [36]. The early-stage release data (≤60% cumulative release) for both R848 and DOX from FA-CS-R848/DOX@Lip were best fitted by the Korsmeyer-Peppas model, while the first-order model provided a comparable fit. These analyses are presented to describe release behavior under the tested conditions rather than to assign a definitive mechanism.

In the cytotoxicity studies, both free DOX and drug-loaded liposomes exhibited significant dose-dependent cytotoxic effects on EO771 cells. Notably, the liposomal formulations displayed markedly superior anticancer activity compared with free DOX, with cell viability reduced across all tested concentrations. This enhanced effect may be attributed to increased intracellular accumulation mediated by active transport mechanisms of the liposomal nanoparticles [34,37]. Compared with R848/DOX@Lip, FA-CS-R848/DOX@Lip showed stronger anticancer efficacy (**p < 0.01), which may result from folate receptor-mediated active targeting that further enhances the selective cytotoxicity toward EO771 cells [38,39]. Moreover, the increased electrostatic interaction between the positively charged FA-CS-R848/DOX@Lip and the negatively charged cell membrane may further promote nanoparticle uptake and improve therapeutic efficacy [40,41].

In the *in vivo* antitumor experiments, the FA-CS-R848/DOX@Lip group exhibited significant tumor growth inhibition. Specifically, tumor volumes in the FA-CS-R848/DOX@Lip group were significantly lower than those in the saline group during the treatment course (****p < 0.0001), supporting robust antitumor efficacy *in vivo*. Beyond the chemotherapeutic effect of DOX itself, this efficacy may also be attributed to the synergistic interaction between DOX and R848. DOX induces ICD, leading to the release of tumor antigens and DAMPs that activate the immune system [42,43]. Meanwhile, R848 was effectively delivered to TAMs, promoting their repolarization from the pro-tumorigenic M2 phenotype to the antitumor M1 phenotype. The drug-loaded liposome group showed a significantly higher MHC II/CD206 ratio in TAMs compared to the control group (****p < 0.0001) as analyzed by flow cytometry, suggesting effective polarization towards the M1 phenotype. Furthermore, the enhanced infiltration and activation of CD8^+^ T cells, as evidenced by elevated IFN-γ and Ki67 levels (****p < 0.0001), represent hallmark features of a robust antitumor immune response. Collectively, these findings suggest that FA-CS-R848/DOX@Lip not only exerts direct cytotoxic effects against tumor cells but also enhances antitumor immunity through immunomodulatory mechanisms.

Despite the encouraging results, several limitations should be acknowledged. First, the use of a single mouse breast cancer model (EO771) may not fully represent the heterogeneity of human breast cancer. Future studies should validate the efficacy of this strategy in other models, including patient-derived xenografts (PDXs). Second, while targeting the FA receptor is effective, the expression levels may vary across different patients and tumor subtypes. Therefore, combining with other targeting ligands, such as CD44 or EGFR receptors, could further enhance specificity and broaden the clinical applicability. Third, the current *in vivo* study compared FA-CS-R848/DOX@Lip only with saline and did not include key control groups such as non-targeted liposomes (e.g., R848/DOX@Lip) or free-drug combinations. Therefore, while our findings support the overall therapeutic potential and feasibility of the targeted multi-agent formulation, they do not enable quantitative deconvolution of the relative contributions from the liposomal carrier, FA-CS targeting, and DOX–R848 synergy. Future work will incorporate these controls to more definitively delineate the roles of active targeting and combinatorial effects. In addition, the specific cell death pathways underlying the antitumor effects were not systematically dissected in this study and warrant further mechanistic investigation. Lastly, the long-term stability of the prepared liposomes and their potential for scale-up in clinical translation require further investigation.

## 5 Conclusion

In conclusion, FA-CS-R848/DOX@Lip, as a multifunctional nanodrug delivery platform, is capable of precisely targeting tumor cells and TAMs. By combining the dual advantages of chemotherapy and immunotherapy, it significantly enhances antitumor efficacy. This platform not only directly induces tumor cell apoptosis but also modulates the tumor microenvironment to enhance the antitumor immune response, highlighting its considerable potential in tumor immunotherapy. Future studies should further explore the long-term therapeutic effects and immune tolerance of this system *in vivo*, and investigate combination therapies with other immunotherapies to further improve its clinical translation potential and therapeutic outcomes.

## Data Availability

Supporting data related to this work are available upon request.
